# Occupational Disparities in the Association between Self-Reported Salt-Eating Habit and Hypertension in Older Adults in Xiamen, China

**DOI:** 10.3390/ijerph13010148

**Published:** 2016-01-21

**Authors:** Manqiong Yuan, Wei Chen, Bogang Teng, Ya Fang

**Affiliations:** 1 State Key Laboratory of Molecular Vaccinology and Molecular Diagnostics, School of Public Health, Xiamen University, Xiang’an Nan Road, Xiang’an District, Xiamen 361102, Fujian, China; yuanmanqiong@163.com (M.Y.); chenw024@163.com (W.C.); 2 Key Laboratory of Health Technology Assessment of Fujian Province University, School of Public Health, Xiamen University, Xiang’an Nan Road, Xiang’an District, Xiamen 361102, Fujian, China; 3 School of Public Health, Xiamen University, Xiang’an Nan Road, Xiang’an District, Xiamen 361102, Fujian, China; tengbg@xmu.edu.cn

**Keywords:** dietary salt intake, hypertension, multivariable logistic regression, occupation, older adults

## Abstract

Blood pressure responses to sodium intake are heterogeneous among populations. Few studies have assessed occupational disparities in the association between sodium intake and hypertension in older people. We used cross-sectional data from 14,292 participants aged 60 years or older in Xiamen, China, in 2013. Self-reported salt-eating habit was examined with three levels: low, medium, and high. The main lifetime occupation was classified into indoor laborer and outdoor laborer. Multivariable logistic regression was used to examine associations of hypertension with self-reported salt-eating habit, main lifetime occupation, and their interactions by adjusting for some covariates, with further stratification by sex. Overall, 13,738 participants had complete data, of whom 30.22% had hypertension. The prevalence of hypertension was 31.57%, 28.63%, and 31.97% in participants who reported to have low, medium, and high salt-eating habit, respectively. Outdoor laborers presented significantly lower prevalence of hypertension than indoor laborers (26.04% *vs.* 34.26%, *p* < 0.001). Indoor laborers with high salt-eating habit had the greatest odds of hypertension (OR = 1.32, 95% CI [1.09–1.59]). An increased trend of odds in eating habit as salt-heavier was presented in indoor laborers (*p-trend* = 0.048), especially for women (*p-trend* = 0.001). No clear trend presented in men. Conclusively, sex-specific occupational disparities exist in the association between self-reported salt-eating habit and hypertension in older individuals. Overlooking the potential moderating role of sex and occupation might affect the relationship between sodium intake and hypertension.

## 1. Introduction

Hypertension has become the leading risk factor of death worldwide [1,2,3,4], especially in China, because of the unprecedented rapid growth of the older population. A recent nationally-representative study showed that more than half of Chinese people aged 60 years or older suffered from hypertension [5], which is responsible for 11.7% of the total mortality [3]. Hypertension is the leading risk factor for heart attack and stroke. An estimated 47% of ischemic heart disease and 54% of stroke events were contributed by hypertension [2]. High blood pressure makes the heart work much harder than it should, but no warning signs appear until the heart has been considerably damaged. Therefore, hypertension is called a “silent killer”. Nevertheless, it can still be prevented by controlling some risk factors.

Excessive sodium is a risk factor for hypertension which has been well documented in epidemiological [6,7] and clinical [8,9] studies. A previous meta-analysis included nine randomized, controlled trials with 14 comparisons between low and normal sodium intake groups [8]. This meta-analysis showed that low sodium intake decreased systolic blood pressure by 0.844 mmHg and diastolic blood pressure by 0.87 mmHg. Some major health organizations, including the United States Department of Agriculture, the Department of Health and Human Services, the Academy of Nutrition and Dietetics, and the American Diabetes Association, provided a consensus recommendation that humans should limit themselves to 1500 mg sodium intake, with no more than 2300 mg per day [10,11]. 

Nevertheless, whether reduction of sodium intake can decrease arterial blood pressure is still controversial [12,13]. The Institute of Medicine has stated that there is insufficient evidence to recommend sodium intake <2300 mg/day in the general population [14]. Additionally, there is evidence that, for some special populations, low sodium intake may be associated with poorer health outcomes [14]. A panel of experts has also reported that potential adverse effects could result from a reduction in sodium intake, such as unhealthy changes in blood lipid and catecholamine levels, and a decline in renal function [15,16,17]. Similarly, a recent study showed that salt intake was not associated with systolic blood pressure [18]. The authors provided an explanation that the link between salt and hypertension was overstated or more complex than once believed. A recent cohort study concluded a J-shaped association between sodium intake and cardiovascular event, and the populations who consume moderate sodium intake (3000–5000 mg/day) have the lowest risk of cardiovascular event that indicated the complex link between sodium intake and blood pressure [19]. Furthermore, some investigators believe that blood pressure responses to alterations in sodium intake are heterogeneous among different populations [20,21]. For example, some studies [22,23] have shown that Black individuals excrete less sodium and have a higher blood pressure after saline administration than White individuals. Therefore, further studies are required to better understand the relationship between sodium intake and hypertension for different populations. 

Sodium in the body mainly comes from salt in the diet and is excreted by sweat and urine to maintain fluid balance. Sweat rates and sodium concentrations have been reported to be related to occupational category [24]. Generally, individuals whose job involves a high air temperature, strenuous physical activity, or a radiant heat source have a high potential to sweat more and this may even lead to excessive sodium loss. Appropriate supplementation of salt is necessary for these individuals. Therefore, we hypothesize that the amount of salt excretion is related to occupational category and, because of this, there may be occupational disparities in the association between sodium intake and hypertension. Additionally, because the effects of sodium intake on the blood pressure regulation are cumulative [25] and dietary sodium consumption can predict future blood pressure [26], the main lifetime occupation instead of current or most recent occupation was used here. In addition, self-reported salt-eating habit has been proved to be an effective predictor of, or a proxy for, actual dietary salt intake [27,28] and, thus, self-reported salt-eating habit was assessed in this study. The present study aims to (1) determine the prevalence of hypertension in older adults in Xiamen and (2) examine the association between dietary salt intake and hypertension in older people, taking into account different types of main lifetime occupation.

## 2. Methods

### 2.1. Study Population 

We carried out a cross-sectional survey among local household-registered adults aged 60 years or older in Xiamen, China, in 2013. To create a baseline database, we proposed to cover 5% of overall registered individuals aged 60 years or older in Xiamen, where there were 261,043 eligible subjects at the time of this survey. Therefore, approximately 13,000 participants were needed. Additionally, for consideration of the effectiveness of returned questionnaires, we expanded our sample size by 10%. Therefore, approximately 14,300 individuals were recruited. This survey took place from 1 August to 2 November in 2013, and the participants were enrolled by a multi-stage sampling procedure. In the first stage, sub-districts in Xiamen (38 sub-districts in total) were all selected. In stage two, one third of communities were randomly sampled from each sub-district and a total of 173 communities were finally included. Randomization of these communities was performed by computer-generated random numbers. In stage three, participants were conveniently recruited from each community by controlling for sex and age composition. The number of individuals to be sampled in each community was determined according to its proportion of eligible older adults. Questionnaires included basic demographic characteristics, activities of daily living, physical health, psychological health, and social support. Questionnaires were completed by face-to-face interview. Interviewers read the questions exactly as they appeared on the questionnaire. Choices of options were verbally provided by the participants and the corresponding codes were then ticked in the questionnaires by the interviewers. Finally, a total of 14,292 questionnaires were recovered. This study was approved by the ethical review committee of the School of Public Health, Xiamen University. Written informed consent was obtained for each participant in the first page of the questionnaire.

### 2.2. Measurements

**Figure 1:**
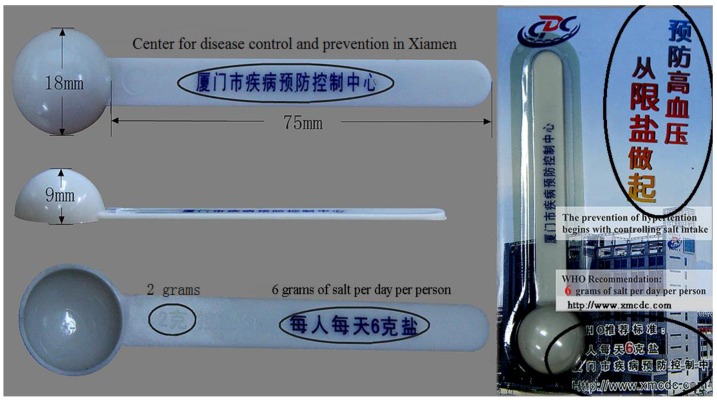
Teaspoon which was promoted by Center for Disease Control and Prevention (CDC) in Xiamen and used to assess the dietary salt intake. There were approximately 2 g of salt in one teaspoon, and CDC in Xiamen recommended that individuals should limit the salt intake to less than 6 g/day (three teaspoons). Chinese characters were circled and the translations were put beside them.

The primary outcome in the present study was the status of hypertension. This was obtained by the question of “Do you suffer from the following physician-diagnosed chronic diseases? (check all that apply)”. Hypertension was the first option among the listed chronic diseases. If this option was ticked, we assumed that the individual suffered from hypertension. Self-reported hypertension has been verified to be highly correlated with physician’s records [29] and its reliability and validity has been examined [30]. The exposure of interest was daily dietary salt intake, represented by self-reported salt-eating habit, was assessed by the question of “What is your daily dietary habit?”, and three options were provided: low, medium and high. Before the participants answered this question, the interviewers showed them a teaspoon, which was promoted by the Center for Disease Control and Prevention (CDC) in Xiamen (Figure 1). There were approximately 2 g of salt in one teaspoon, and the CDC in Xiamen recommends that individuals should limit salt intake to less than 6 g/day (three teaspoons/day), which approximately equates to 2300 mg of sodium. If the participant took three or less teaspoons of salt per day, they were suggested to answer the questionnaire as “low”. If they took three to nine teaspoons per day, they were “medium” and if took more than nine teaspoons, they were “high”. We hypothesized that the main lifetime occupation was a modifier in the association between dietary salt intake and hypertension, because the sweat rates and sodium concentrations in sweat were related to the occupational type [24]. Occupation was classified into two categories, which were indoor labor (including office worker in government, non-government and private company, production line worker, waiter, housekeeping, individual merchant, housewife, and homemaker) and outdoor labor (including farming, fishing, forestry, construction worker, and others who work in the open air). Outdoor laborers were supposed to have a high sweat rate and excrete more sodium than indoor laborers. Additionally, some demographic characteristics (age, sex, body mass index [BMI], marriage, education, and the number of children), and life habits (smoking, alcohol drinking, and exercise) were considered as covariates. Among them, BMI was classified into four categories according to the BMI cut-off points for Chinese: <18.5 kg/m^2^ for underweight, (18.5–22.9) kg/m^2^ for normal weight, (23.0–26.9) kg/m^2^ for overweight, and ≥27 kg/m^2^ for obese [31]. Smoking (alcohol drinking) was measured by the question that “How often do you smoke cigarettes (have drinking containing alcohol)?”. Exercise was measured by the question that “How often do you do physical activities for health (not including activities for work)?”. Since sex-specific differences in hypertension have been frequently addressed, we further stratified the analyses by sex.

### 2.3. Statistical Analysis

First, we summarized the characteristics of participants by their status of hypertension using descriptive statistics. Percentage distributions of participant for dietary salt intake in each occupational category were also provided. The Chi-square test (for categorical variable) or Cochran-Armitage trend test (for ordinal variables) was used to assess the associations between various factors and hypertension status, and also to evaluate the association between self-reported salt-eating habit and occupation. A *p*-value < 0.05 was considered statistically significant. Second, multivariable logistic regression (Equation (1)) was performed to model the associations of hypertension with self-reported salt-eating habit, occupation, and their interactions, adjusting for the covariates mentioned above.

*logit* [Probability(*H*)] ~ *Occupation + Salt + Occupation × Salt + Covariates* (1)

where *H* represented hypertension status, *Occupation* represented main lifetime occupation, *Salt* was the self-reported salt-eating habit, and *Covariates* included sex, age level, education, BMI, marriage, number of children, smoking, alcohol drinking, and exercise. Finally, we stratified the regression by sex. Odds ratios (ORs) and corresponding 95% confidence intervals (CIs) were estimated, where the indoor laborer who reported to have low salt-eating habit was the reference category. The trends of odds ratio in three levels of salt intake were evaluated by treating it as a continuous variable, *i.e.*, coding low, medium and high salt-eating habit as 0, 1, and 2, respectively. All the analyses were performed using R script. More specifically, the logistic regression was performed using the function of *glm*() in the package of “stats” and the ORs when accounted for interactions were estimated by the function of *glht*() in the package of “multcomp” in R script.

## 3. Results 

### 3.1. Hypertension Prevalence 

Among the 14,292 recovered questionnaires, 213 had missing data on one or more adjustment variables and 341 were long term jobless, and both were excluded. Table 1 shows the characteristics of the 13,738 participants according to the status of hypertension. The overall prevalence of hypertension was 30.22%. Women presented significant higher prevalence of hypertension than men (*p* = 0.044). As age advanced, the prevalence of hypertension increased (*p* < 0.001), and we found that the average age was higher in hypertensive participants than in those who were not hypertensive (*p <* 0.001). Although self-reported salt-eating habit was associated with hypertension status (*p* < 0.001 for Chi-square test), but the trend was not significant (*p* = 0.976 for Cochran-Armitage trend test). Moreover, multiple comparison tests indicated that individuals who reported to have medium salt-eating habit had lower prevalence of hypertension than people with low or high salt-eating habit, with false discovery rate adjusted *p* values 0.006 and 0.032, respectively. Indoor laborers presented significantly higher prevalence of hypertension than outdoor laborers (*p <* 0.001). Unexpectedly, attainment of a higher education appeared to be related to older adults being at a disadvantage in controlling blood pressure as older people with a higher education had greater prevalence of hypertension compared with those with a lower education (*p <* 0.001). Participants who had more children showed higher prevalence of hypertension (*p =* 0.003). Conclusively, sex, age, salt-eating habit, main lifetime occupation, education, number of children, smoking, and alcohol drinking were significantly associated with hypertension status (*p <* 0.05). Table 2 depicted the percentage distributions of participant for self-reported salt-eating habit in each occupational category stratified by sex. Most (>90%) of the participants reported to have low or medium salt-eating habits. The percentages of high salt-eating habit were higher for men than women (9.38% *vs.* 6.18%, *p <* 0.001). The Chi-square tests indicated that self-reported salt-eating habit were associated with the occupational category (*p <* 0.001).

**Table 1:** Basic characteristics of 13,738 participants according to the status of hypertension.

Characteristic	Normotension	Hypertension	Prevalence ^a^ (%)	*p * ^b^
**Total, N**	9586	4152	30.22	
**Sex, N (%)**				0.044
Female	5033 (52.5)	2258 (54.38)	30.97	
Male	4553 (47.5)	1894 (45.62)	29.38	
**Age, mean(SD)/years**	70.92 (8.34)	72.91 (8.21)	-	<0.001
**Age level, N (%)**				<0.001
60~	2751 (28.7)	790 (19.03)	22.31	
65~	2211 (23.06)	858 (20.66)	27.96	
70~	1508 (15.73)	759 (18.28)	33.48	
75~	1416 (14.77)	786 (18.93)	35.69	
80~	961 (10.03)	575 (13.85)	37.43	
85~	739 (7.71)	384 (9.25)	34.19	
**Salt-eating habit, N (%)**				<0.001/0.976 **^d^**
Low	4283 (44.68)	1976 (47.59)	31.57	
Medium	4584 (47.82)	1839 (44.29)	28.63	
High	719 (7.5)	337 (8.12)	31.91	
**Occupation, N (%)**				<0.001
Indoor labor	4591 (47.89)	2393 (57.63)	34.26	
Outdoor labor	4995 (52.11)	1759 (42.37)	26.04	
**Education, N (%)**				<0.001
Illiterate	3230 (33.69)	1295 (31.19)	28.62	
Primary	3033 (31.64)	1169 (28.16)	27.82	
Junior high school	1763 (18.39)	838 (20.18)	32.22	
Senior high school and beyond	1560 (16.27)	850 (20.47)	35.27	
**BMI (kg/m^2^) ^c^, N (%)**				0.132
Underweight (<18.5)	477 (4.98)	189 (4.55)	28.38	
Normal (18.5–22.9)	5733 (59.81)	2453 (59.08)	29.97	
Overweight (23.0–26.9)	2735 (28.53)	1239 (29.84)	31.18	
Obese (≥27)	641 (6.69)	271 (6.53)	29.71	
**Marriage, N (%)**				0.079
In-marriage	6730 (70.21)	2826 (68.06)	29.57	
Single	98 (1.02)	42 (1.01)	30.00	
Divorced	96 (1)	49 (1.18)	33.79	
Widowed	2662 (27.77)	1235 (29.74)	31.69	
**Number of Children, N (%)**				0.003
0	246 (2.57)	84 (2.02)	25.45	
1	1482 (15.46)	578 (13.92)	28.06	
2	2518 (26.27)	1112 (26.78)	30.63	
≥3	5340 (55.71)	2378 (57.27)	30.81	
**Smoking, N (%)**				<0.001
Never	6024 (62.84)	2832 (68.21)	31.98	
Sometimes	1365 (14.24)	489 (11.78)	26.38	
Often	1660 (17.32)	526 (12.67)	24.06	
Quit	537 (5.6)	305 (7.35)	36.22	
**Alcohol Drinking, N (%)**				<0.001
Never	6469 (67.48)	2896 (69.75)	30.92	
Sometimes	2279 (23.77)	861 (20.74)	27.42	
Often	491 (5.12)	193 (4.65)	28.22	
Quit	347 (3.62)	202 (4.87)	36.79	
**Exercise, N (%)**				0.057
Never	3602 (37.58)	1472 (35.45)	29.01	
Sometimes	3505 (36.56)	1581 (38.08)	31.09	
Often	2479 (25.86)	1099 (26.47)	30.72	

**^a^** prevalence of hypertension; **^b^** *p* value of Chi-square test or Cochran-Armitage trend test to assess the relationship between hypertension and the other variables. For the ordinal variables including age, salt-eating habit, education, BMI, and number of children, Cochran-Armitage trend test was used, and for the others, Chi-square test was used; **^c^** BMI: body mass index; **^d^** Both Chi-square test or Cochran-Armitage trend test were used, obtaining *p* value < 0.001 and *p* value = 0.976, respectively.

**Table 2:** Percentage distributions of participant for self-reported salt-eating habit in each occupational category stratified by sex.

Salt-Eating Habit	Occupation, N (%)		*p* ^a^
	Indoor	Outdoor	
**Male (*N* = 6447)**			<0.001
Low	1201 (45.46)	1377 (36.19)	
Medium	1179 (44.63)	2085 (54.80)	
High	262 (9.92)	343 (9.01)	
**Female (*N* = 7291)**			<0.001
Low	2281 (52.53)	1400 (47.47)	
Medium	1784 (41.09)	1375 (46.63)	
High	277 (6.38)	174 (5.90)	

**^a^***p* value of Chi-square test to assess the association between dietary salt intake and occupation

### 3.2. Interactions of Occupation and Self-Reported Salt-Eating Habit on Hypertension

**Figure 2:**
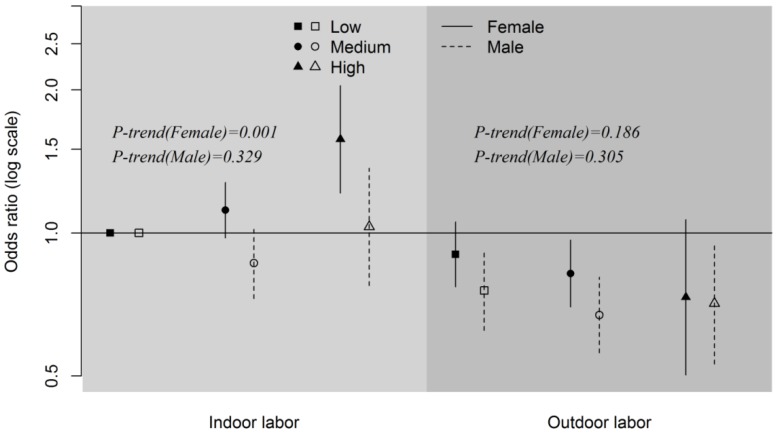
Sex-stratified odds ratios with corresponding 95% confidence intervals of joint association of self-reported salt-eating habit and main lifetime occupations on hypertension. The symbols indicate point estimates of odd ratios and the vertical bars indicate the corresponding 95% confidence intervals. Indoor laborers who reported to have low salt-eating habit in both sexes were the reference categories. *p-trend* was *p-value* of trends for odds ratio for three levels of self-reported salt-eating habit.

Table 3 shows the ORs with corresponding 95% CIs for joint association of occupation and self-reported salt-eating habit on hypertension. Indoor laborers who reported to have low salt-eating habit were the reference category. Among all of the groups, indoor laborers with high salt-eating habit had the greatest odds of hypertension (OR = 1.32, 95% CI [1.09–1.59]). ORs of hypertension for outdoor laborers were obviously lower than indoor laborers at all three levels of salt-eating habit. Participants who consumed more dietary salt had higher odds of hypertension for indoor laborers (*p-trend* = 0.048), but no clear trend was presented for outdoor laborers (*p-trend* = 0.091). ORs with corresponding 95% CIs stratified by sex are shown in Figure 2. Overall, female participants appeared to have a greater variation in ORs under the same occupational category than male participants, which may suggest that women were more salt sensitive. Additionally, an increased trend of odds as eating habit was salt-heavier presented in female indoor laborers (*p-trend* = 0.001), but no such trend, or even an opposed trend despite not statistically significant (*p-trend* = 0.186), was shown for female outdoor laborers. No clear trend presented in men. Moreover, almost all of the ORs presented higher for women than men in participants who were engaged in the same occupational category and simultaneously had the same level of salt-eating habit. Outdoor laborers showed lower ORs than indoor laborers for both men and women.

**Table 3:** Odds ratios (ORs) with 95% confidence intervals (CI) obtained from multivariable logistic regression **^a^**.

Occupation	Low		Medium		High		*p*-trend ^b^
	OR	95% CI	OR	95% CI	OR	95% CI	
Indoor labor	1	-	1.01	0.91–1.13	1.32	1.09–1.59	0.048
Outdoor labor	0.84	0.75–0.95	0.75	0.67-0.85	0.75	0.60-0.94	0.091

**^a^** Estimates were adjusted sex, age, salt-eating habit, occupation, education, body mass index, marriage, number of children, smoking, alcohol drinking, and exercise; **^b^** *p-trend*: *p*-value of trends for odds ratio for three levels of self-reported salt-eating habit.

## 4. Discussion

There is a growing consensus of opinion that a reduction in dietary salt intake has favorable effects in controlling blood pressure. Numerous studies have shown the salt sensitivity is heterogeneous among different populations [20,21]. While most research on hypertension has focused on some non-modifiable factors, such as sex [32], genes [33,34], and race [35,36], involvement of occupational disparities in the association between dietary salt intake and hypertension has been poorly investigated. Different OR patterns among occupations may reflect the importance of occupational mediation. In this study, varied OR patterns were observed in outdoor laborers and indoor laborers. We found that female indoor laborers were most salt-sensitive, with significantly higher odds of hypertension when they reported to have higher salt-eating habits. However, such a disadvantage was not significant in outdoor laborers and male indoor laborers.

Our study indicated that the joint association of self-reported salt-eating habit and main lifetime occupation on hypertension was more pronounced in women than in men. Some previous studies [37,38] have also reported that the blood pressure response to dietary salt intake is stronger in women than in men. Such differences may be due to the effect of female sex hormones, such as estrogen and progesterone, which are thought to be related to sodium reabsorption and water retention [39,40]. Men might also excrete more salt (approximately 0.3 g/day) than women through urine [41] which, in turn, makes men less salt sensitive. Additionally, in a previous study [42], a significant interaction between perceived stress and occupation was observed in women only. Perceived stress then contributes to elevated blood pressure. Similarly, a recent study on the effect of working hours and hypertension in Koreans showed that there was a higher hazard ratio of hypertension in female workers than male workers [43]. The authors explained that women have to bear the burden of a job and domestic work, and moreover, the working burden is higher for female workers than male workers.

In the present study, the prevalence of hypertension was higher in women than men. Although pre-menopausal women have a lower rate of hypertension than age-matched men, their blood pressure elevated much faster after menopause [44]. The women in this study were probably in their post-menopause years as they were all aged 60+ years. The mechanisms by which blood pressure increases in postmenopausal women were not completely understood, but there are some possibilities: (1) post-menopausal women exhibit enhanced activation of the renin-angiotensin system, an important regulator of blood pressure and fluid and sodium balance, and this contributes to salt retention and the evolution of hypertension [45]; (2) women were found to have a tendency to gain weight during the menopause [46], which makes them more prone to high blood pressure; (3) anxiety and depression are common symptoms of the menopause [47], which may also negatively impact on the blood pressure [48]; (4) post-menopausal women secrete slightly more testosterone than premenopausal women [49], and this hormone may also contribute to elevated blood pressure by altering a number of humoral factors [50,51]. 

In the current study, outdoor laborers showed a slightly decreased trend in the odds of hypertension when they reported to have higher salt-eating habits, although the trend was not significant (*p-trend* = 0.091). They perform a lot of physical activity during work and, thus, likely have a higher sweat rate. Therefore, they may excrete more sodium. Proper supplementation of salt can regulate blood pressure in a healthy manner [52]. Moreover, occupation can directly affect the individual’s blood pressure through many other ways, such as working circumstances, working hours, and stress [42,53,54]. A previous study suggested that working with high noise levels significantly elevated blood pressure [53]. Individuals who work 40 h per week were 14% more likely to suffer from hypertension than those who work between 11 and 39 h per week [55]. 

Education has been well demonstrated as a protecting factor for hypertension in developed countries [56]; however, a reverse gradient result was presented in this study. Illiterate participants showed the lowest prevalence of hypertension (28.62%) while those who received senior high school or beyond showed the highest one (35.27%), with a *p*-value of trend <0.001. A similar result was also found in populations in other developing countries, such as in Indians [57], Cuban men [58], Mexican women [59], and Jamaican men [60]. Some have argued the reverse association could be caused by dietary factors. Higher-educated participants always have a higher socioeconomic status and, thus, may have higher income. However, among low-income populations, higher socioeconomic status may be used to purchase “unnecessary” calories and, thus, increase the risk of hypertension [59]. Another possible causation explaining the positive relationship between higher education and hypertension could be that more educated people had higher rates of self-reported hypertension while less educated people may have never heard of hypertension.

## 5. Limitations of the Study

To the best of our knowledge, this is the first study to investigate the modification of occupation on the association between dietary salt intake and hypertension. There are a number of strengths in this study, including using recent, large-scale samples and stratifying the modification of occupation by sex. Nevertheless, some limitations should also be acknowledged. First, despite a large sample size, the findings were based on a cross-sectional survey in a single city. Salt-eating habits are regionally characterized and, therefore, our results may be localized and, thus, limit the applicability of the findings throughout the country. Previous study has revealed that mean northern sodium intake was 4733 mg/person/day, significantly higher than the southern 2491 mg/person/day [61]. Moreover,the Xiamen CDC has pointed out that Xiamen residents usually have a light diet, and the average daily salt intake per person is 8–10 g (approximately 3.14–3.93 g sodium). Second, the status of hypertension was obtained by self-reported questionnaires and only physician-diagnosed hypertensive participants were assumed to have hypertension. Therefore, we may have underestimated the prevalence of hypertension. However, self-reported hypertension was highly correlated with physician’s records [29] and its reliability and validity had been examined [30]. Moreover, for medical screening purposes, people who were aged 60 years or older in Xiamen can participate in an annual physical examination for free in recent years, including checking blood pressure. Third, the measurement of dietary salt intake was rough and relatively subjective. Recently, inaccuracy of self-reported low sodium diet among Chinese has been reported [62]. However, the samples in that study were all from Shangdong, a province in Northern China where people consume significantly more sodium than subjects in Xiamen [61]. Due to the higher average sodium intake, people in Northern China probably have higher standard level of self-reported salt intake than Southern China and, therefore, the conclusion that inaccuracy of a self-reported low sodium diet may merely be applicable for Northern China. In addition, some articles have also pointed out that self-reported taste preference or salt-eating habit can be a proxy for daily sodium intake [27,28]. To further confirm the relationships among occupation, sex, sodium intake, and hypertension, studies using objective or quantitative measurements, such as blood pressure and urinary sodium excretion, are urgently required. Fourth, changes in salt dietary habits were not tracked. People may change their dietary habits in a healthy direction if they realize the effects of salt intake on health. Tracking dietary habits of salt intake can effectively and accurately assess its cumulative effect on hypertension.

## 6. Conclusions

In summary, there are sex-specific occupational disparities in the association between self-reported salt-eating habit and hypertension. Female indoor laborers have the highest salt sensitivity. A reduction in dietary salt intake may not have advantages in preventing hypertension among outdoor laborers. Moreover, older women have a higher prevalence and greater variation in the ORs of hypertension. Therefore, more detailed guidelines of sodium administration according to specifications of an individual are required. Finally, to accurately confirm the relationships among sex, occupation, dietary salt intake and hypertension, studies using objective or quantitative measurements are required.
